# Neuroprotective Strategies during Cardiac Surgery with Cardiopulmonary Bypass

**DOI:** 10.3390/ijms17111945

**Published:** 2016-11-21

**Authors:** Aida Salameh, Stefan Dhein, Ingo Dähnert, Norbert Klein

**Affiliations:** 1 Clinic for Paediatric Cardiology Heart Centre, University of Leipzig, 04289 Leipzig, Germany; ingo.daehnert@medizin.uni-leipzig.de; 2 Rudolf-Boehm-Institute for Pharmacology and Toxicology, University of Leipzig, 04107 Leipzig, Germany; stefan.dhein@medizin.uni-leipzig.de; 3 Department of Cardiology, Angiology and Internal Intensive Care Medicine, St. Georg Hospital, Academic Medical Centre, University of Leipzig, 04129 Leipzig, Germany; norbert.klein@sanktgeorg.de

**Keywords:** cardiopulmonary bypass, neuroprotection, heart-lung machine

## Abstract

Aortocoronary bypass or valve surgery usually require cardiac arrest using cardioplegic solutions. Although, in principle, in a number of cases beating heart surgery (so-called off-pump technique) is possible, aortic or valve surgery or correction of congenital heart diseases mostly require cardiopulmonary arrest. During this condition, the heart-lung machine also named cardiopulmonary bypass (CPB) has to take over the circulation. It is noteworthy that the invention of a machine bypassing the heart and lungs enabled complex cardiac operations, but possible negative effects of the CPB on other organs, especially the brain, cannot be neglected. Thus, neuroprotection during CPB is still a matter of great interest. In this review, we will describe the impact of CPB on the brain and focus on pharmacological and non-pharmacological strategies to protect the brain.

## 1. Introduction

At the end of the 19th century, it was widely accepted among surgeons that the heart should not be touched. In 1898 the famous physician Billroth said in his legendary quotation, that a surgeon who wants to maintain the respect of his colleagues should never dare to perform heart sutures [1]. Until the middle of the 20th century, cardiac operations if they were ever performed were accompanied by a high mortality rate. This situation changed due to the development of the heart lung machine cardio-pulmonary bypass (CPB) by John Gibbon Jr. and surface cooling by Wilfred Gordon Bigelow [2,3]. Both inventions had been a major advancement in cardiac surgery and established our modern heart surgery. Although, some cardiac operations like bypass-surgery can be carried out on the beating heart using the off-pump technique (i.e., without CPB), for most inborn cardiac malformations, aortic surgery, or valve replacement the heart lung machine is indispensable. Thus, the development of CPB was a real blessing for cardiac surgery but possible negative impacts on the perfused internal organs should not be neglected. The brain, an organ highly sensitive to hypoxia, is threatened by thromboembolic ischemic stroke, hemorrhage, or inflammation during CPB. Moreover, cognitive impairments like memory deficits, concentration difficulties, or impaired fine-motor skills have been described after CPB [4,5].

Remarkably, depending on the patient collective, up to 50% of the patients suffered from these symptoms [6]. Although neurological deficits after cardiac operations are mostly subclinical and long term outcome is usually good, they may interfere with daily life and may compromise patient activity. At least three types of neurophysiological impairment can be discriminated: (a) a more general deficit without focal symptoms regarding intellectual properties/performance and memory, which is assumed to be the result of a global hypoperfusion affecting the watershed regions (in particular, the hippocampus) and might result in neurological deficit in up to 50% of the patients [7]; and (b) more focal symptoms related to thromboembolism, mainly in the cerebri media area. The latter occurs with incidences ranging from 1.9% to nearly 10%, depending on the cardiac operation which was carried out (CABG (coronary artery bypass graft) alone, CABG together with valve surgery, single, double or triple valve surgery) and can be reduced by the use of filters [8,9]. However, other studies on the question of whether or not CPB leads to cognitive disturbances came to a different view. In these studies, on-pump (with CPB) operated patients were compared with off-pump operations and it became evident that, several months after the surgical procedure, only marginal differences in the neurological outcome existed, which were completely absent in a follow-up after one year [10,11]. In another study, a decreased myocardial injury was seen in the off-pump group but again no differences in the neurological outcome [12]. In their excellent review on the on-pump off-pump debate Kennedy et al. [13] drew the same conclusion.

Therefore, it was concluded that CPB was not the cause for the described neurological deficits and that neuropsychological alterations occurred in the on-pump as frequently as in the off-pump group, which argues against a causal connection between neurological decline and CPB. Indeed, during off-pump operations, manipulation of the aorta or luxation of the heart with subsequent impaired right ventricular filling and low left ventricular output might also deteriorate neurological function and might counteract potential advantages of this operation technique. Moreover, it might be that more severe cases were operated with CPB and less severe cases with the off-pump technique which also affects study outcome. In an analysis of Cheng et al. [14] including 37 randomized studies, the authors did not find any differences in the occurrence of myocardial infarction, stroke, renal failure, and mortality between the on-pump and the off-pump groups. However, the authors stated that a lack of statistical significance did not necessarily mean that both operation techniques are equal, they declared that it rather shows an insufficient study power to prove whether there are differences or not. Indeed, the reasons for a neurological deficit after heart operations are multifactorial and not easy to assess. Additionally, clinical studies on this issue are difficult to compare since CPB is not a well-standardized technique with important variabilities between and also within different institutions. Thus, the final words on neurological disturbances after CPB have not yet been spoken and neuroprotection during CPB or in general during heart operations still remains a matter of special interest.

It has been known for a long time that CPB initiates a cascade of inflammatory reactions. The reason for this is the contact of patient blood with external surfaces (tubes, connections, membrane oxygenator, etc.) of the heart-lung machine. As a result, activation of the complement system, of platelets, leucocytes, and endothelial cells occur which in turn are responsible for the release of various pro-inflammatory cytokines such as IL-6, IL-8, IL-10, or TNFα [15]. This systemic inflammatory response syndrome (SIRS) might then result in a leakage of the blood-brain-barrier and in brain edema. Moreover, thromboembolism, fat embolism, air bubbles, and embolism of atherosclerotic plaques are not uncommon during cardiac surgery. Although, bubble traps and filtration of the arterial line minimize thromboembolism, the risk of emboli mobilization during manipulation of the aorta remains [16].

Additionally, CPB was developed with a laminar flow profile which is not physiological, as in normal circulation a pulsatile flow is present. This pulsation might be necessary to maintain a sufficient microcirculation and to supply the internal organs with oxygen and nutrients. Regarding the blood supply of the brain, it is known that a tight autoregulation exists which keeps cerebral blood flow constant within 50–150 mmHg of arterial blood pressure [17]. Nevertheless, in studies of Kusch et al., and Özatik et al. (Özatik 2002) it was shown that serum S100beta levels were higher in patients operated using non-pulsatile CPB compared to CPB with pulsatile flow, and Özatik et al. concluded that lower levels of S100β are the result of lower systemic inflammation during pulsatile CPB (S100β which belongs to the S100 protein family, serves as a Ca^2+^-binding protein and has been proposed as a biomarker for neuronal cell damage) [18,19]. However, pro-inflammatory cytokines such as IL-6 and IL-8 were not different between both patient groups (pulsatile versus non-pulsatile CPB). Therefore, these data do not proof that pulsatile flow indeed reduces inflammation.

On the other hand, systemic inflammation can be suppressed pharmacologically and there are hints that application of corticosteroids might be of advantage during CPB in lowering NSE (neuron specific enolase, also a marker for cerebral injury) and S100β protein release, although this is still a matter of debate [20]. Whether suppression of inflammation also has a protective effect on the neurological outcome has yet to be proven. Furthermore, it is important to mention that (despite an appropriate cardiac output delivered by the heart-lung machine) the brain is threatened by hypoperfusion at the beginning of CPB, which may be related to low mean arterial pressure during cannulization and manipulation of the great vessels or the heart and which might critically reduce blood flow in watershed regions. Moreover, reperfusion at the end of CPB causes another pathophysiological situation with enhanced free radicals.

What do we know about the involved mechanisms finally leading to cerebral cell death during ischemia/reperfusion injury? Once the O_2_-level drops HIF (hypoxia inducible factor)—which is a key factor in regulating oxygen homeostasis—translocates into the nucleus. HIF consists of α- and β-subunits with the α-subunit being constitutively expressed but rapidly degraded under normal oxygen concentrations. Decreasing oxygen pressure causes stabilization of the α-subunit which then dimerizes with the β-subunit and acts as a transcription factor for various genes involved in cell survival [21]. These genes include for example VEGF (vascular endothelial growth factor) required for angiogenesis, genes necessary for erythropoietin production or for glucose utilization [22,23,24,25]. On the other hand, it was described that HIF1α has also negative aspects regarding cell protection. Several authors found that HIF1α induces apoptosis: based on an animal study of Althaus et al. on transient cerebral ischemia, the authors could demonstrate that pro-apoptotic factors such as BNIP3 are upregulated by HIF1α, which finally caused neuronal cell death [26]. Moreover, in a model of embryonic stem cells it was shown that HIF1α reduced hypoxia-dependent proliferation and induced apoptosis whereas inactivation of HIF1α caused a decrease in apoptosis rate [27]. These results were confirmed by a more recent study of Dai et al., who demonstrated enhanced apoptosis in a human pancreatic cell line when HIF1α was upregulated by hypoxic conditions [28]. Thus, whether HIF1α has more protective or more detrimental properties still has yet to be evaluated and also might be tissue-dependent.

Moreover, another interesting protein which is upregulated by hypoxic conditions was described in two recently published studies by Mohammed Abdul et al. [29,30]: the SUR2A (sulfonylurea receptor 2A) protein. SUR-proteins are regulatory elements of the K_ATP_-channel. These ATP (adenosine triphosphate) sensitive potassium channels are composed of SUR1, SUR2A, or SUR2B subunits together with K_ir_6.1 or K_ir_6.2 depending on the tissue where they are expressed. In the heart, it was demonstrated that the expression of SUR2A is increased under hypoxic conditions and that this increase was accompanied by an improved survival of cardiomyocytes [29,30]. In the brain, the subunit SUR1 is co-expressed with K_ir_6.x, and in experimental models of stroke it was demonstrated that blockade of SUR was associated with reduced infarct size and reduced cerebral edema [31,32]. A decrease in oxygen is accompanied by a collapse of the respiratory chain and thus a decline in ATP-levels. Due to a failure of ATP-dependent ion pumps, intracellular sodium and calcium levels rise which results in cytoplasmic and mitochondrial swelling which at the end might lead to cell death. The cellular calcium overload itself is accompanied by an activation of several proteases and phospholipases which impair the integrity of cell membranes [33]. Moreover, elevated calcium levels might initiate mitochondrial permeability transition with a loss of function and with release of AIF (apoptosis-inducing factor) and cytochrome c. Both molecules are involved in programmed cell death. In general, there are two main ways for apoptosis initiation: the caspase-dependent and the caspase-independent pathway. AIF, a factor located behind the outer membrane of the mitochondrion, is released during mitochondrial permeability transition. AIF then translocates into the nucleus thereby initiating apoptosis. This is the caspase-independent intrinsic pathway and results in DNA fragmentation [34]. The caspase-dependent apoptotic pathway (extrinsic pathway) is started by cytochrome c release. Cytochrome c is located at the inner mitochondrial membrane and is involved in the electron transport process. Once cytochrome c is released after mitochondrial damage it activates caspase-9 which in turn activates the caspase-cascade initiating DNA condensation and cell apoptosis. On the other side, depending on the phosphorylation status of cytochrome c, anti-apoptotic actions are also possible [35]. Moreover, apoptosis can be inhibited by several other proteins such as HSP70 (heat shock protein 70), Bcl-2 (B-cell lymphoma 2) or BCL-xL (B-cell lymphoma-extra-large) [36,37]. In the brain, a special situation exists: in hypoxic or ischemic conditions the neurotransmitter glutamate is released by neuronal cells resulting in excitotoxicity and finally in neuronal cell death [38]. Additionally, activation of glutamate receptors may increase intracellular sodium and calcium levels, which also is deleterious for neuronal cells.

In the phase of reperfusion reactive oxygen species (ROS) are produced predominantly in mitochondria or peroxisomes and react with NO (derived for example from endothelial cells) to form peroxynitrite. This highly-reactive molecule nitrosylates proteins, thereby changing or impairing their function. The proteins with nitrotyrosine-residues can be detected with anti-nitrotyrosine antibodies and can serve as a marker for oxidative/nitrosative stress. Furthermore, NO is responsible for impaired mitochondrial respiration due to interference of NO with oxygen at cytochrome oxidase. Thus, cells are sensitized against hypoxia, aggravating impaired cellular respiration [39]. On the other hand, low levels of NO are cell protective in the sense that NO inhibits apoptosis, mitochondrial permeability transition and, in the case of the heart, preserves contractile function [40,41].

Furthermore, peroxynitrate induces nuclear DNA strand breaks which have to be repaired as they are fatal for cellular survival. The enzyme PARP (poly-ADP ribose polymerase) tags these DNA breaks with PAR (poly-ADP ribose)-strands and thus gives the repair signal for DNA ligases and polymerases [42]. Although this is, in principal, a positive process to maintain cellular function it has on the other side also negative aspects: DNA repair depletes NAD^+^ and ATP stores and might—via an over-activation of PARP—lead to cell death. Furthermore, accumulation of intracellular PAR molecules leads to inhibition of glycolysis (via inhibition of hexokinase) and promotes the release of AIF thus inducing the so-called parthanatos [43]. In consequence, inhibition of PARP might be a useful tool to protect cells against an over-activation of PARP followed by energy depletion and death. In several animal studies (heart and brain), it was demonstrated that inhibition of PARP resulted in less cellular damage, less inflammation, and a better outcome for organs after ischemia and reperfusion injury [44,45].

Another point to address is the activity of matrix metalloproteinases (MMPs). These calcium-dependent endopeptidases are able to degrade compounds of the extracellular matrix such as laminin, collagen, fibronectin, or elastin. They are important in regulating cellular migration, differentiation, apoptosis, and angiogenesis. Up to now, 28 MMPs have been discovered which are initially synthetized as inactive zymogenes. After cleavage of a pro-peptide site the MMP is activated and ready for cleavage of connective tissue [46]. Conversely, tissue inhibitors of metalloproteinases (TIMPs) negatively regulate MMP activity. However, there is some evidence that TIMPs might induce increased deposition of extracellular matrix irrespective from their inhibitory action on MMPs [47].

In the brain, the loss of blood-brain-barrier is associated with MMP activity: in several animal studies on ischemic stroke a role for MMPs (MMP-3, -9, -12) in the development of hemorrhage after ischemic insult has been proposed [48,49]. Additionally, since more than two-thirds of all strokes are of the ischemic type, the patients would benefit from thrombolysis with tPA (tissue plasminogen activator), the most-widely used medicament for this therapy. Nevertheless, this drug also activates MMP-9 resulting in central edema, hemorrhagic complications, increased infarct size, and poor outcome [50]. During inflammation, MMPs are released and as we know that CPB induces inflammatory processes, it was not far to seek for an influence of MMPs during CPB. In a patient study on about 90 patients undergoing CABG (coronary artery bypass grafting) Kotlinkska et al. demonstrated that, during bypass, serum levels of MMP-9 were significantly increased which was accompanied by significant increases in S100β protein and CK-BB (creatine kinase isoenzyme BB) and interestingly was positively correlated with enhanced jugular venous bulb pressure [51]. The authors then concluded that enhanced central venous pressure impaired blood-brain-barrier with enhanced serum S100β and CK-BB levels thereby promoting brain edema.

In summary, it can be stated that brain protection during CPB is of special interest and that different pharmacological and non-pharmacological methods have been tested during the setting of CPB. Although some concepts and approaches are applied in the operation theatre, most of the pharmacological protection strategies have only been tested in animal experiments.

## 2. Apparative Methods for Brain Protection

In the year 1952, Cooley and DeBakey described various cases of aneurysmatic aortic lesions and their surgical therapy [52]. The main problem of these operations is the circulatory arrest which is needed for aortic repair. Indeed, neurological complications are not rare—literature data vary between 5% and 25%, and most importantly the rate of neurological complications is positively correlated with the patient’s age and the duration of circulatory arrest [53,54]. Thus, new methods to better preserve the brain had to be sought. Lowering of body core temperature via the CPB and additional surface cooling prevented damage of the brain and spinal cord and extended the time of cardiac arrest. However, disagreement still remains to which extent body core temperature has to be lowered: some surgeons prefer higher hypothermic temperatures (20–28 °C) whereas some surgeons use deep hypothermia of 10–13 °C during aortic repair. In their review, Griepp and Di Luozzo summarized metabolic rates of the brain at various temperatures and the safety duration of circulatory arrest and it became obvious that the lower body temperature meant less metabolic activity of the brain could be measured and the longer the predicted safety interval for surgery was [55]. However, the disadvantages of deep hypothermia should not be neglected. Depending on the body temperature and on the modalities of re-warming organ dysfunction like renal failure, postoperative bleeding, or even rhabdomyolysis may occur. Depending on the complexity of aortic surgery the time gained with hypothermia might not be sufficient and thus, additional selective brain perfusion (antegrade or retrograde) during body cooling was introduced and several studies compared the mode of brain perfusion and temperature management. It became apparent that the best strategy for neuroprotection was the combination of hypothermia with selective antegrade head perfusion, whereas the degree of hypothermia (above or below 20 °C) remains to be discussed [56,57,58]. However, it should be noted—as described above—that the perfusion flow, generated by the CPB is a non-pulsatile flow which is in contrast to the physiological pulsatile flow generated by the heart. Although brain perfusion remains constant within a wide range of blood pressure values due to auto-regulative processes, there is some evidence that a more physiological (i.e., pulsatile) flow might be of advantage. In a recently published manuscript Zhao et al. a clear advantage of pulsatile flow was described with regard to oxygen saturation as well as an improvement of microcirculation in a study comprising 40 infants with Tetralogy of Fallot undergoing cardiac surgery with either pulsatile or non-pulsatile CPB [59]. A possible disadvantage of the pulsatile roller pumps might be a higher level of hemolysis and possible platelet activation [59]. In contrast, others did not find advantageous effects of pulsatile flow during CPB [60,61]. However, it should be emphasized that studies on this issue are difficult to compare as the performance of CPB is not a standardized procedure and the number of patients included in these studies is low.

In a study of our working group piglets, of about 10 kg were evaluated with pulsatile and non-pulsatile bypass and it became evident that especially the kidneys and hippocampus profited from the pulsatile flow modality in such a way that markers of hypoxia, oxidative stress, and apoptosis were much lower in tissues exposed to the pulsatile flow [62]. Thus, it might be worth evaluating the advantages (or disadvantages) of a pulsatile flow in larger patient cohorts with a standardized regimen. Since the perfusion deficit mainly seems to affect the hippocampal region, which is a watershed region, and is more likely to represent a hypoperfusion rather than thromboembolism, follow-up of these patients needs to include cognitive tests and memory assessment.

As described above, CPB initiates an inflammatory response with activation of leucocytes, platelets, endothelial cells, the coagulation system, as well as fibrinolysis. These potentially adverse influences on a patient’s outcome could be minimized by using (for example) leucocyte filters, heparin coated circuits, or tubes coated with certain polymers [63]. A recently published study compared the beneficial effects of leucocyte depletion in combination with polymer-coated tubes and it was demonstrated that patients of this study arm compared to control patients had a significantly reduced risk of postoperative bleeding, atrial fibrillation, less need for catecholamine therapy, and a shorter period of mechanical ventilation [64]. Renal or neurological complications were slightly but not significantly reduced. However, other study groups did not observe a beneficial effect of leucocyte reduction on post-operative outcome [65]. The reason for these contradictory results might be related to different patient population (i.e., different morbidity) or different severity of cardiac operation or to statistical problems as a lack of power.

Another interesting point to discuss is the influence of perfusion pressure on neurological outcome. In a retrospective study with over 1000 patients, the influence of mean arterial pressure dispersion during CPB was analyzed and it became evident that besides other factors like age, duration of CPB, or known cerebrovascular diseases, patients with high arterial pressure fluctuations had the highest incidence of stroke development [66]. In an animal study with piglets, Walther et al. systematically evaluated the effects of different flow rates and temperature on mean arterial pressure, jugular and regional oxygen saturation, and histological markers of brain damage [67]. In this experimental work, the authors could demonstrate that the reduced mean arterial pressure during hypoperfusion led to reduced carotid blood flow, reduced tissue oxygen saturation, increased lactate, and malonedialdehyde levels and histological damage of the hippocampus. Moreover, in the hypothermia groups, brain damage was less compared to the normothermic groups regardless whether moderate hypothermia (25 °C) or deep hypothermia (18 °C) was administrated [67]. In another study on 30 human patients undergoing cardiac surgery with CPB cerebral blood flow was not different under reduced pump flow conditions (1.2 L/min/m^2^) compared to normal perfusion (2.3 L/min/m^2^) unless mean arterial pressure was kept constant [68].

Besides perfusion pressure and flow rate, the amount of oxygen carrier is also essential to provide the tissue with O_2_. The normal hematocrit value is between 40%–54% for men and 36%–48% for women. During CPB hemodilution is normally accepted which reduces the amount of transfusion with red blood cells on one hand and also improves microcirculation on the other hand [69]. However, hemodilution might also be disadvantageous as brain oxygen supply might be reduced if hematocrit is too low. This problem was addressed by a very recent study of Del Felice et al. [70], who examined the influence of different hematocrit levels on cerebral EEG (electroencephalogram). From this study and others, it became evident that a hematocrit level of about 25% was not associated with an impaired outcome [71].

In summary, to definitely evaluate the beneficial effects of different perfusion techniques during CPB—i.e., pulsation, priming solution, temperature, perfusion pressure, and flow etc.—larger clinical studies with long-term neurophysiological follow-up have to be realized to evaluate protective effects on the brain.

**Figure 1:**
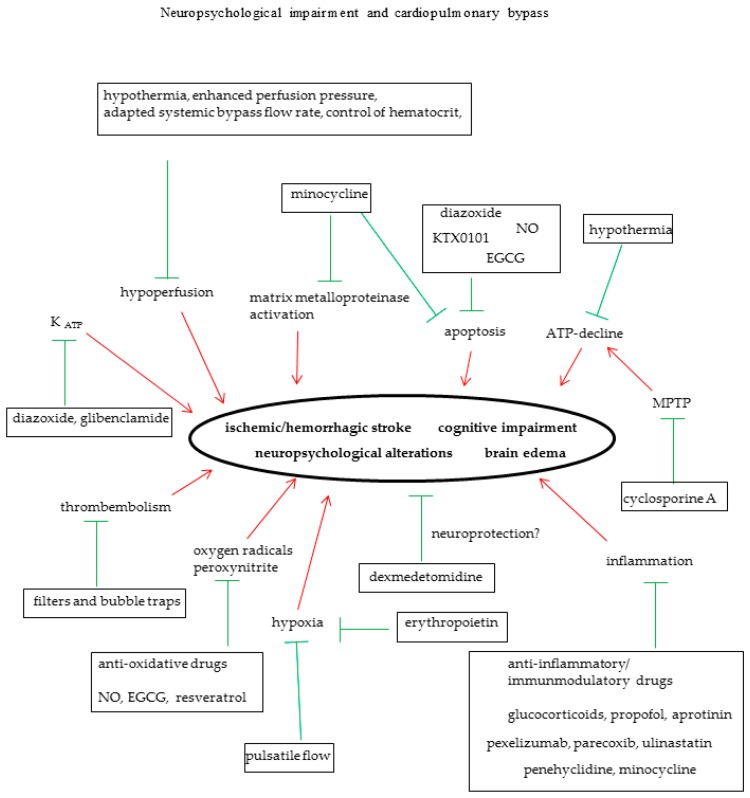
Pathophysiology of neurological deficits in the course of cardiopulmonary bypass and possible pharmacological and non-pharmacological interventions. Red arrows indicate negative effects on the brain, green lines show protective strategies. EGCG, epigallocatechin-3-gallate; NO, nitric oxide; K_ATP_, ATP-sensitive potassium channel; MPTP, mitochondrial permeability transition pore.

Figure 1 summarizes methods for neurological protection.

## 3. Pharmacological Methods for Brain Protection

### 3.1. Anti-Inflammatory Drugs

As described above CPB initiates an inflammatory response which might negatively affect post-operative neurological outcome. One of the oldest drugs against inflammation are the glucocorticoids and already 25 years ago they were applied during cardiac surgery [72]. It was found that prednisolone or its methylated derivative significantly altered various cytokine concentrations such as interleukin-4, -6, -8, -10, or TNFα [73,74].

However, the effect of glucocorticoids on CPB-induced inflammation was discussed controversially. On one hand, due to the complexity of inflammatory responses not all inflammatory mediators can be suppressed by glucocorticoids even at high dosages and on the other hand the clinical outcome of patients often is not positively influenced despite a sufficient suppression of inflammation [74,75,76]. In a recently published study of our working group conducted in pediatric patients with inborn cardiac malformations it was demonstrated that, even though methylprednisolone reduced pro-inflammatory responses, a clear benefit regarding post-operative outcome was not detected [77]. However, it might be that patients with complex cardiac operations requiring long machine times would benefit from glucocorticoid treatment. Interestingly, an older report described significant cerebral edema (in six of six patients) immediately after cardiac surgery (CABG) which subsequently subsided and which was not accompanied by neurological deterioration [78]. In a more recent study, these results could not be confirmed. Moreover, it was demonstrated in another trial with over 4000 patients that dexamethasone reduced post-operative infection but did not influence post-operative stroke rate and—as a negative side effect—was associated with elevated blood glucose levels [79,80].

Other drugs with expected immune modulatory effects have also been tested: the anesthetic propofol had a positive influence on the immune response after CPB [81]. Furthermore, it was found that S100β levels were lower in patients receiving propofol in contrast to patients under desflurane anesthesia [82].

Other anti-inflammatory drugs include the serine protease inhibitor aprotinin or complement inhibition by pexelizumab. Both treatments during CPB showed less inflammation in the treatment group and especially pexelizumab seems to have a positive effect on cardiac injury. However, neurological outcome was only assessed in one study on dogs where the authors could demonstrate significantly decreased S100β levels when aprotinin was added to the priming solution [83,84]. Moreover, the selective COX2 (cyclooxygenase 2)-inhibitor parecoxib has also been tested in patients undergoing CPB and it was shown that pro-inflammatory cytokines were significantly lower and anti-inflammatory cytokines significantly higher in the parecoxib group [85]. The authors could also demonstrate that myocardial injury assessed by troponin T levels was significantly less in the parecoxib arm. However, post-operative outcome and especially cerebrovascular complications were not different in both groups.

The trypsin inhibitor, ulinastatin, also reduced the inflammatory response and exhibited neuroprotective properties in a piglet model of low-flow CPB [86]. Especially, histological examination of the hippocampus showed less cellular injury. However, in a patient study reported by Qiu et al. [87], ulinastatin exhibited no positive effects on post-operative outcome. Similarly, the anesthetic ketamine which also has been identified as an anti-inflammatory drug did not show any neuroprotective properties [88].

Taken together, inhibition of inflammation might be a useful approach but whether neurological outcome really is improved by this intervention still has to be evaluated.

Some other promising drugs have been discovered but, to date, are used only in animal experiments. Anti-inflammatory action was attributed to the recently described anticholinergic drug penehyclidine. This drug was developed in 2005 and antagonizes the muscarinergic and nicotinergic effects of acetylcholine [89]. In a rat model of CPB, pretreatment with penehyclidine showed a significant reduction of markers for neuronal injury, inflammation, and apoptosis. Moreover, electron microscopy evaluations revealed that the occurrence of damaged mitochondria was significantly lower in the penehyclidine group [90].

In addition, studies from our working group and from others demonstrated that the tetracycline derivative minocycline significantly attenuated neuroinflammation caused by cardiac arrest or CPB [91,92]. In these studies, minocycline was able to reduce neuronal TNFα-levels and to inhibit hypoxic and apoptotic cell lesions. Additionally, it significantly reduced cellular edema. Figure 1 summarizes anti-inflammatory drugs for brain protection.

### 3.2. Miscellaneous Drugs

Since the discovery of an endothelium-derived relaxing factor (EDRF) by Furchgott and Zawadzki in the year 1980 and the confirmation by Ignarro et al., that it was nitric oxide (NO) a lot of research had been done around this interesting molecule [93,94]. Recent studies on patients evaluated the influence of NO given during CPB. These studies demonstrated an anti-oxidative, anti-apoptotic, and cardio-protective effect of NO with less troponin and BNP (brain natriuretic peptide) release and less need for diuretics. However, markers of inflammation were not altered by NO and a possible benefit for the brain has not been assessed [95,96]. Since NO-donors or NO inhalation is used for treatment of coronary sclerosis and treatment of pulmonary hypertension, it may be possible that the vasodilating properties of NO might also be beneficial for brain perfusion.

Furthermore, anti-oxidative properties have been attributed to EGCG (epigallocatechin-3-gallate), one of the typical ingredients of green tea. Our working group examined the influence of CPB and EGCG on hippocampi of piglets and we could show that pericellular edema, transcription factors like HIF1α or AIF, as well as cleaved caspase 3 were significantly attenuated in the EGCG group [91]. Also, other organs like kidney and lung profited from EGCG [97,98,99]. However, EGCG has not yet been tested in a clinical setting of CPB.

Another molecule with interesting properties is the sodium salt of d-β-hydroxybutyrate, namely KTX0101. β-hydroxybutyric acid is a naturally occurring compound and is synthesized in the liver from acetoacetate. It is elevated during fasting and it can be used as an alternative energy source by the brain [100]. There is some evidence that KTX0101 might be beneficial in ischemia/reperfusion injury, for the treatment of Alzheimer’s disease and—as has been postulated—it might also protect the brain during CPB [101,102,103]. However, currently there are no studies available demonstrating positive effects of KTX0101 during CPB.

Another protection principle is the use of K_ATP_ openers. K_ATP_ channels have been found in many cell types including hippocampal mitochondria. Several years ago, it was demonstrated in an experimental model of cortical ischemia that the K_ATP_ opener diazoxide completely prevented neuronal cells from ischemic damage [104,105]. In another study of Shake et al., this medication was tested in dogs undergoing a 120 min hypothermic circulatory arrest [106]. After rewarming and disconnection from CPB, the animals were tested regarding their neurological outcome and after slaughter the brain was examined histologically. The results of this study were as follows: diazoxide significantly improved the neurological outcome of the dogs in such a way that ataxia and other coordination abnormalities fully recovered after 72 h. Moreover, histological staining of hippocampus and cerebellum revealed that the control animals (CPB without diazoxide) had more signs of cellular apoptosis, inflammation, or necrosis compared to the diazoxide-treated animals. Co-administration of a selective mitochondrial K_ATP_ blocker together with diazoxide abolished the beneficial effect of the latter one. However, these promising results have not been confirmed in a clinical study.

In contrast, the K_ATP_ blocker glibenclamide, an anti-diabetic drug, has also been tested in animal models of stroke and it could be demonstrated that glibenclamide improved neurological outcome and prevented neuronal death and cerebral edema in the infarcted region [31,32]. However, during CPB in clinical settings K_ATP_ blockers have not been tested so far.

Anti-oxidative drugs like resveratrol and MPTP (mitochondrial permeability transition pore) blockers like cyclosporine A have also been tested in various brain ischemia models and it was demonstrated that both drugs exerted beneficial effects [107,108]. Nevertheless, they have not been used during CPB so that a potential effectiveness during CPB has not been proven.

Dexmedetomidine, an α_2_-adrenoceptor agonist with sedative and presumably neuroprotective properties, showed a reduction of postoperative delirious phases but had no influence on neurological outcome [109]. Other drugs like erythropoietin have been tested in small patient studies demonstrating less cognitive impairment of the patient group receiving erythropoietin before CPB [110]. These positive results have been overshadowed by another study which showed a higher rate of death in the erythropoietin group [111]. However, patient collectives of both studies differ significantly: on one hand, patients with no neurological disorders undergoing CABG and on the other patients with ischemic stroke. Thus, if erythropoietin could be safely administered during CPB, it still has to be evaluated in larger clinical trials [112]. Figure 1 summarizes medical strategies for improvement of neurological outcome.

## 4. Summary

Neuroprotective techniques are still a matter of interest in cardiac surgery, since cardiopulmonary bypass can cause neurological damage either by (a) thromboembolism; (b) hypoperfusion of watershed regions; or (c) inflammatory processes. Current neuroprotective approaches include non-pharmacological and pharmacological strategies. Thus, besides anticoagulation, filters and bubble traps have been employed to avoid thromboembolic stroke. Hypothermia has been used to lower the metabolic rate of the neurons thereby making the cells less vulnerable to hypoxic damage. Microcirculation might be improved and hypoperfusion might be prevented by a more physiological flow profile (e.g., by pulsatile flow). Regarding pharmacological strategies anti-inflammatory and anti-ischemic approaches can be distinguished. Anti-inflammation can be achieved with systemic glucocorticoids and with the trypsin inhibitor ulinastatin. For anti-ischemic therapies, drugs are required which can pass the blood-brain barrier and can protect neurons against ischemia/reperfusion injury. Among these drugs EGCG and minocycline act as radical scavengers and anti-apoptotic agents. The latter also exerted anti-inflammatory actions by inhibition of TNFα. Another approach uses K_ATP_ opener (such as diazoxide) to protect neurons, presumably by hyperpolarization. From a theoretical point of view, activation of excitotoxic NMDA receptors (*N*-methyl-d-aspartate receptors) in ischemia/reperfusion has been discussed as a possible target of neuroprotection. However, experiments with the NMDA-antagonists, memantine and ketamine, did not show neuroprotection in circulatory arrest [88,113].

Regarding clinical studies, prospective controlled investigations with long-term neurophysiological follow-up are still rare, so that final statements on neuroprotective efficacy are difficult. The quest for effective neuroprotective drugs during CBP is not at its end but still at the beginning.
